# A thrombolytic therapy using diagnostic ultrasound combined with RGDS-targeted microbubbles and urokinase in a rabbit model

**DOI:** 10.1038/s41598-020-69202-9

**Published:** 2020-07-27

**Authors:** Lina Guan, Chunmei Wang, Xue Yan, Liyun Liu, Yanhong Li, Yuming Mu

**Affiliations:** Department of Echocardiography, First Affiliated Hospital of Xinjiang Medical University, Urumqi, Xinjiang People’s Republic of China

**Keywords:** Endocrinology, Endocrine system and metabolic diseases

## Abstract

This study aimed to explore thrombolysis therapy based on ultrasound combined with urokinase and Arg–Gly–Asp sequence (RGDS)-targeted microbubbles by evaluating the histological changes in a thrombotic rabbit model. Forty-two New Zealand rabbits featuring platelet-rich thrombi in the femoral artery were randomized to (n = 6/group): ultrasound alone (US); urokinase alone (UK); ultrasound plus non-targeted microbubbles (US + M); ultrasound plus RGDS-targeted microbubbles (US + R); RGDS-targeted microbubbles plus urokinase (R + UK); ultrasound, non-targeted microbubbles and urokinase (US + M + UK); and ultrasound, RGDS-targeted microbubbles and urokinase (US + R + UK) groups. Diagnostic ultrasound was used transcutaneously over the thrombus for 30 min. We evaluated the thrombolytic effect based on ultrasound thrombi detection, blood flow, and histological observations. Among all study groups, complete recanalization was achieved in the US + R + UK group. Hematoxylin and eosin staining showed that the thrombi were completely dissolved. Scanning electron microscopy examination demonstrated that the fiber network structure of the thrombi was damaged. Transmission electron microscopy showed that the thrombus was decomposed into high electron-dense particles. Histology for von Willebrand factor and tissue factor were both negative in the US + R + UK group. This study revealed that a thrombolytic therapy consisting of diagnostic ultrasound together with RGDS-targeted and urokinase coupled microbubbles.

## Introduction

Thrombosis is responsible for myocardial infarction and ischemic stroke, which are associated with high mortality and morbidity. The common endpoint in these pathophysiological conditions is the formation of arterial thrombi.

Urokinase activates plasminogen found on the surface and within thrombi and converts it into plasmin, which in turn, dissolves the thrombus by cleaving the fibrin protein matrix, leading to the dissolution of the thrombi and release of visible components including red blood cells and platelets^1^. Urokinase thrombolysis treatment is less intrusive and logistically easier compared to percutaneous intervention and surgery. Nevertheless, practically, urokinase treatment achieves a lower initial recanalization rate than interventional or surgical methods, and is associated with a high incidence of reocclusion and systemic bleeding events that may threaten one’s life, and could lead to worse short- and long-term clinical consequences^2^.

We have studied and tested in conjunction with thrombolytic agents to facilitate thrombus disruption in patients cavitational and non-cavitational effects of ultrasound (US) in order to overcome the limitations of conventional urokinase therapy^3–5^. Recent studies have shown that the mechanisms responsible for the augmentation of thrombus lysis are an increase in magnitude and depth of penetration of drugs into the thrombus, as well as a greater exposure of binding sites to various drugs due to the effects of US on the thrombus^6–8^. Low power US not only increases microbubble destruction but also serves as the driving force for microbubbles acting on the surface of thrombus^9,10^.

Microbubbles are generated as US contrast agents, which are encapsulated gas bubbles and drugs, which can accelerate US thrombolysis^11^. Possible mechanisms, at least partially, may involve an enhancement of acoustic cavitation or oscillation of microbubbles^12–14^. Nonlinear bubble activity, which occurs within the vasculature upon US exposure, leads to cavitation, which can impose mechanical stress on nearby cells and junctions^15^, which has been believed to cause cavitational threshold lower and microstreaming higher^16–19^.

Thrombolysis, improved through ultrasound-mediated method is regarded to be mainly mechanical in nature^20^. Acoustic streaming, created by absorbing the ultrasonic energy^21^, boosts drug penetration into thrombi^22^ while the acoustic force displaces the thrombus^23^. Featuring gentle nonlinear oscillations of bubbles, acoustic emissions from stable cavitation^24^ are associated with the enhancement of drug thrombolysis^13,14^. Microstreaming is induced by these stable bubbles^25^, and is believed to help to increase penetration of drugs into the thrombus^14^. Microstreaming is also deemed to detach fibrin-degradation products, so as to make plasminogen binding sites more available for urokinase^26^.

The platelet membrane receptor IIb/IIIa (GP IIb/IIIa) has a specific recognition and binding site, the Arg-Gly-Asp sequence (RGDS)^10^. Activated platelets are targeted and bound to RGDS-modified liposomes, resulting in platelets aggregation^27^, suggesting the feasibility of developing a delivery system of platelet-targeted anti-thrombogenic drug. Studies on thrombolysis by ultrasound combined with microbubble contrast agent and urokinase has been proven in animal models to be a better alternative therapy to drug alone or surgical intervention^28^. Nevertheless, the mechanisms of the thrombolysis therapy and in vivo histological changes are still poorly understood.

Therefore, we hypothesized that diagnostic ultrasound combined with urokinase and RGDS-targeted microbubbles may be a safe and efficient thrombolysis therapy.

## Methods

### Preparation of microbubbles

Urokinase (prourokinase, Nanjing Nanda Pharmaceutical Co. Ltd., Nanjing, China) was labeled with FITC (fluorescein isothiocyanate, Chinese Peptide Company, Hangzhou, China). The (RGD) peptides were labeled with 5-carboxytetramethylrhodamine (5-TAMRA, GL Biochem Ltd., Shanghai, China), by electrostatic adsorption based on the manufacturer’s instructions. The microbubbles carrying urokinase were prepared as previously described^29^. Briefly, 3 mg of 5-TAMRA-conjugated RGDS (5-TAMRA-RGDS), 3 mg of FITC-conjugated pro-urokinase (FITC-UK), and 3 mg of SonoVue (BRACCO Imaging B.V., Geneva, Switzerland) were dissolved in 1 × PBS, to a final volume of 6 ml and a final microbubble concentration of approximately 1 × 10^9^ microbubbles/ml. Urokinase and RGD were conjugated by acoustic vibration and then allowed to standby for 30 min at 37 °C. The particle size was analyzed by a cytometer (CYTOMETER, Beckman, USA).

### Rabbit model of acute femoral artery embolism

Forty-two New Zealand rabbits (male and female), weighing 1.8–2.8 kg, were acquired from the animal center of Xinjiang Medical University, Urumqi, China. The rabbits were provided with sufficient food and water and acclimated for 1 week before the experiments.

Heparin (1,000 U/kg) was bolus injected intravenously before thrombolytic therapy. The rabbits were anesthetized (pentobarbital, 30 mg/kg followed by 10 mg at 30- to 60-min intervals, via marginal ear vein injection). A 23-G catheter was inserted into the marginal ear vein for fluid maintenance. An incision was made in the femoral artery region. Femoral arteries were dissected 5 cm distal to the origin of the superficial branch, and the profound and superficial femoral arteries were ligated. A Doppler flow probe (Model TS420, Transonic System, Ithaca, NY, USA) was placed distally around the isolated segment to monitor blood flow. An occlusive suture was placed around the artery, and the distal clamps were loosened after 7 to 8 min. Occlusive thrombi were formed within 20 to 30 min, as expected, in all femoral arteries. Occlusive thrombi were detected by two-dimensional and color Doppler imaging. B-mode imaging showed the presence of low-echo-level thrombi over the local femoral artery, indicating that no blood flow, pulse signal, or microbubbles were present. The endpoint of thrombolytic therapy was set at 120 min after treatment^30^.

Rabbits were then randomized to (n = 6/group): ultrasound alone (US); urokinase alone (UK); ultrasound plus non-targeted microbubbles (US + M); ultrasound plus RGDS-targeted microbubbles (US + R); RGDS-targeted microbubbles plus urokinase (R + UK); ultrasound, non-targeted microbubbles and urokinase (US + M + UK); and ultrasound, RGDS-targeted microbubbles and urokinase (US + R + UK) groups. The solution (3 ml) containing RGDS, microbubbles, and urokinase was administered to the rabbits as an intravenous bolus at 5 min duration, and 3 ml of the same solution solution were injected continuously over 20 min via the marginal ear vein. Then, the rabbits were subjected to contrast pulse sequencing to visualize the femoral artery. The MI was increased to 1.0 and applied at a frame rate of 3.1 MHz along the long axis plane. The MI was then turned back to 0.08, and images were captured again.

The animals were used according to the Guide for the Care and Use of Laboratory Animals. The experimental procedures were approved by the Ethics Committee of Xinjiang Medical University (No. 20090317001).

### Ultrasound

The ultrasound examination was performed with a GE Vivid Seven (GE Medical Systems, Little Chalfont, UK). A high-frequency i13L transducer (5.7–11.4 MHz, GE Medical Systems) was used for B-mode and color Doppler imaging. The output power was set at − 15 dB, the mechanical index (MI) at 0.13, and the depth at 3 cm. The GE Vivid Seven ultrasound system with an M3S transducer (3.5 MHz, GE Medical Systems) was used for flashing the microbubbles. The mechanical index (MI) was adjusted according to the low-MI setting (0.08 MI with contrast pulse sequence) for imaging microbubbles of the femoral artery and high-MI (1.0) for diagnostic application. The transducer was fixed in a longitudinal view above a coupling medium tank 3–4 cm in depth so that the ultrasound beam could provide adequate coverage for the potential area of thrombosis. The rabbits were examined in the supine position.

### Evaluation of the thrombolytic effect

The thrombi were extracted at room temperature and centrifuged at 2000–3,000 rpm for 20 min. A microtiter plate was coated with HRP-conjugated rabbit urokinase antibodies (Beyotime, China). Urokinase was added to the plate to form antigen–antibody and enzyme-antibody complexes. Tetramethylbenzidine (TMB) was added for reading the wells. The urokinase activity was measured using a microplate reader (Bio-Rad, USA) and determined by the equation of Urokinase = OD (optical density) × (− 2.3971 + 13.81172 X × 0.995748). The concentration of urokinase was measured at baseline and at 10-min intervals until the end of the experiment. The residual urokinase was dispersed in blood and metabolized via liver and renal.

### Histology

The animals were sacrificed by overdosing with pentobarbital (900 mg/kg) at the end of the experiment. Then, the proximal and distal ends of the arteries were ligated and excised. The contralateral femoral artery was examined as a control. The arteries were immersed in 4% paraformaldehyde (overnight) and embedded in paraffin. Samples were sectioned to 3–4 μm, mounted, and stained with hematoxylin and eosin (H&E). Sections were scanned for histological changes under a microscope (Olympus BX43, Olympus Corporation, Tokyo, Japan). Skeletal muscle and its surrounding tissues were subjected for H&E staining.

### Fluorescence microscopy, scanning electron microscopy (SEM) and transmission electron microscopy (TEM)

Transverse 4-μm thick sections of arteries and smears of arterial blood were observed under fluorescence microscopy (Carl Zeiss GmbH, Oberkochen, Germany), using the contralateral femoral artery as control. The femoral artery was cut from the midportion of the thrombus-containing vessel and fixed in 4% glutaraldehyde for SEM and TEM analyses. The samples were dried with a critical-point dryer using liquid CO_2_ and isoamyl acetate, mounted, and sputter-coated with platinum for SEM observation. The samples were examined and photographed using a JSM-6301F electron microscope (JEOL Ltd., Tokyo, Japan).

### Evaluation of the thrombolytic effect

Doppler flowmetry was used to assess thrombosis and thrombolysis according to the methods described previously^31^. Briefly, the occlusive thrombi in all the femoral arteries showed a blood flow velocity < 0.5 ml/min on the Doppler flow examination. Two-dimensional imaging showed the presence of a low-echo-level thrombus over the femoral artery. No blood flow, pulse signal, or microbubbles were detected by color and pulsed Doppler imaging. The endpoint of thrombolytic therapy was set at 120 min after drug treatment.

### Immunohistology staining

The procedure of immunohistology staining has been narrated in previous paragraph^32^. The antibodies for TF and vWF were all from Abcam (Cambridge, UK), and the dilution was 1:200.

### Analysis of statistics

All statistical analyses were conducted using SPSS 16.0 (IBM, Armonk, NY, USA). Data are shoen as means ± standard deviation (SD) and were analyzed using one way analysis of variance (ANOVA) and the Student–Newman–Keuls (SNK) post hoctest. *P* < 0.05 was considerd statistically significant.

## Results

### Appearance and optical microscopic observations of the contrast agent

**Figure 1:**
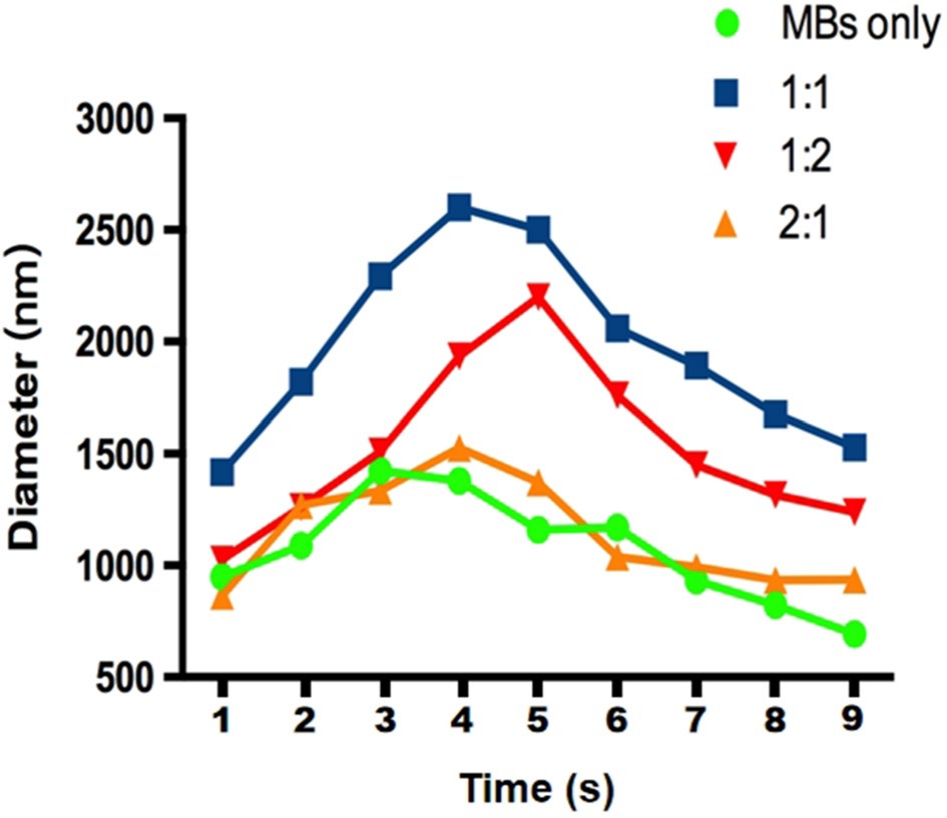
The particle size distribution of each group of contrast agents. Green dots: blank contrast agent Sonovue. Blue squares: 1:1 ratio of urokinase/RGDS. Red downward triangles: 2:1 ratio of urokinase/RGDS. Orange upward triangles: urokinase/RGDS 1: 2 ratio of prepared contrast agent.

The targeted contrast agents were pink, curd-like, opaque, and easy to delaminate after standing, in which the upper layer was white and contained curd-like microbubbles, and the lower layer consisted of a mildly opaque, translucent pink liquid. The sizes and concentrations were 1915 ± 457.8 nm and 3.36 × 10^8^ microbubbles/ml, respectively, and the binding rate of urokinase/RGDS was the highest among the groups (Table 1, Fig. 1).

**Table 1:** The size of the particles.

	n	Size (nm)	*P*
Control	9	1,077.1 ± 233.1	
1:1	9	1915 ± 457.8^▲^	0.019
2:1	9	1,126.4 ± 281.6	
1:2	9	1647 ± 420.1^▲^	

^▲^Compared with control, ^▲^*p* < 0.05.

### US + R + UK and R + UK achieved better thrombolytic effects

The US, US + M, US + R, UK, or US + M + UK groups showed no recanalization during the 120 min after treatment, while the US + R + UK and R + UK achieved recanalization (Table 2).

**Table 2:** Comparison of femoral arterial blood flow over 120 min (ml/min).

	Blood flow						Sum	F*	P*
	10	20	30	60	90	120			
US	3.96 ± 5.21	1.93 ± 2.66	4.95 ± 7.81	4.63 ± 7.16	1.01 ± 1.35	2.67 ± 5.73	3.19 ± 5.29	0.670	0.649
US + M	5.51 ± 4.28	3.33 ± 3.22	2.67 ± 2.61	1.24 ± 1.05	0.84 ± 0.28	0.50 ± 0.28	2.35 ± 2.89	1.354	0.268
UK	8.92 ± 9.65	4.60 ± 3.27	3.97 ± 2.74	0.50 ± 0.40	0.43 ± 0.43	0.38 ± 0.24	3.13 ± 5.10	4.220	0.005
US + M + UK	3.24 ± 1.95	3.82 ± 5.64	1.48 ± 1.21	4.42 ± 6.54	4.06 ± 6.78	2.20 ± 3.01	3.20 ± 4.51	0.826	0.541
US + R	0.50 ± 0.88	0.68 ± 0.57	0.47 ± 0.47	0.13 ± 0.20	0.41 ± 0.37	0.11 ± 0.04	0.38 ± 0.50	0.020	> 0.99
R + UK	4.57 ± 2.52	6.67 ± 9.42	10.50 ± 1.27^&,▲,Ф,#,*^	14.67 ± 3.98^&,▲,Ф,#,*^	9.53 ± 2.73^&,▲,Ф,#,*^	8.10 ± 1.56^&,▲,Ф,#,*^	9.00 ± 5.28	3,383	0.015
US + R + UK	3.29 ± 4.97	7.55 ± 6.35	9.37 ± 6.50^▲,Ф,#,*^	13.09 ± 4.28^&,▲,Ф,#,*^	17.27 ± 4.50^&,▲,Ф,#,*,♦^	17.10 ± 4.51^&,▲,Ф,#,*,♦^	11.28 ± 6.89	11.355	< 0.001
Sum	4.28 ± 4.97	4.08 ± 5.32	4.78 ± 5.30	5.53 ± 6.88	4.80 ± 6.74	4.44 ± 6.48	4.64 ± 5.95		
F*	1.79	1.320	5.117	11.645	22.817	25.101		F^#^ = 3.732	
*P* < 0.001	P*	0.13	0.274	0.001	< 0.001	< 0.001		< 0.001	

^&^P versus US at the same time point; ^▲^P versus US + M at the same time point; ^Ф^P versus UK at the same time point; ^#^P versus US + M + UK at the same time point; P versus US + R at the same time point; ^♦^P versus R + UK group at the same time point.

**Figure 2:**
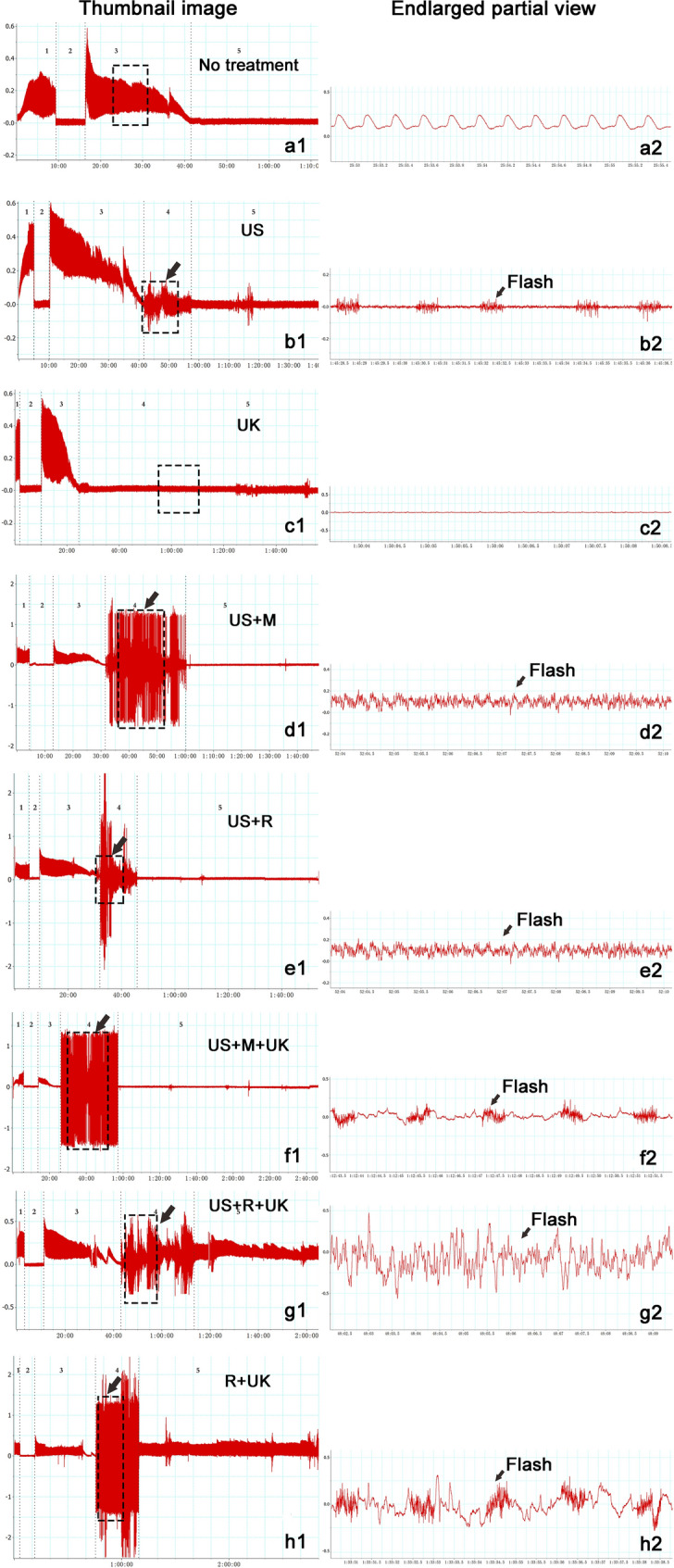
Record of blood flow and wave shape in representative cases. Right side: thumbnail image, Left side: enlarged partial view. The experiment was divided into 4–5 periods according to different stages of treatment in thumbnail image. 1, Baseline: No treatment. 2, Occlusion time: Occlusive suture was placed around the artery, and there was no flow for 7 to 8 min. 3, Thrombus time: The distal clamps were loosened and occlusive thrombi were formed within 20 to 30 min in femoral arteries. 4, Thrombolysis time: different thrombolytic methods were used after forming a stable occlusion. 5, Observation time: the thrombolytic effect was observed after treatment. Black arrow: Using ultrasound to flash.

Thirty minutes prior to the observation of thrombolysis, a large, high-amplitude wave was observed in the R + UK and US + R + UK groups (Fig. 2), which also led to a higher and larger resonance wave after flashing by US in the US + R + UK groups (Fig. 2).

No significant difference in wave shape was observed among the US, US + M, US + R, and US + M + UK groups. In addition, a small, low-amplitude stray wave was sustained, with no resonance after US flashing in the US, US + M, US + R, and US + M + UK groups (Fig. 2). Moreover, urokinase concentration in the UK and R + UK groups were significantly different at 90 min compared to baseline (*P* < 0.05). Urokinase concentration of the US + M + UK and US + R + UK groups showed a noticeable drop at 30 min and a statistically significant change at 60 min compared to baseline (*P* < 0.05).

**Figure 3:**
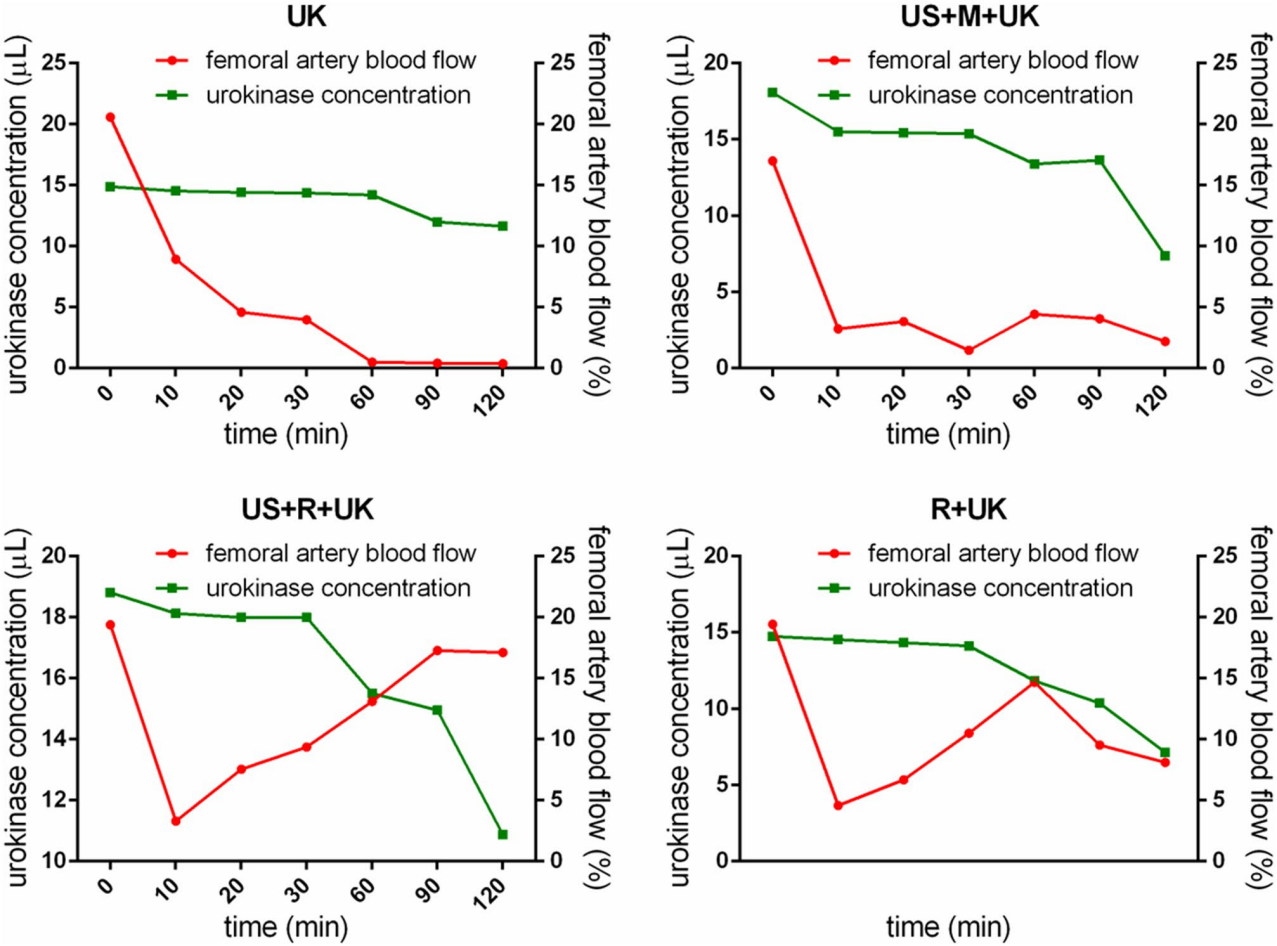
Urokinase concentration and blood flow. The urokinase concentration of the R + UK and US + R + UK group dropped to the lowest point at 60 and 100 min, respectively, while the blood flow peaked at the same time points. UK: urokinase alone; US + M + UK: ultrasound, non-targeted microbubble and urokinase; R + UK: RGDS-targeted microbubble plus urokinase; US + R + UK: ultrasound, RGDS-targeted microbubble and urokinase. Squares indicate the serum concentration of urokinase. Circles indicate the femoral artery blood flow.

Furthermore, the urokinase concentration of the R + UK group dropped to the lowest point at 60 min, while the blood flow recovered to its peaked (Fig. 3 and Table 3). The same trend was also observed in the US + R + UK group, however the major difference between the two pattems was that they reached the lowest point at different time points (60 min for R + UK vs. 100 min for US + R + UK). Supplementary Figure S1 presents a summary of the B-mode and color Doppler ultrasound examinations in the absence of thrombus, with complete occlusion, at reocclusion, and during recanalization.

**Table 3:** Comparison of the serum concentration of rabbit urokinase.

Time (min)	UK	US + M + UK	R + UK	US + R + UK
	n = 6	n = 6	n = 6	n = 6
0 (baseline)	11.63 ± 5.64	13.98 ± 4.12	13.77 ± 3.08	18.12 ± 2.19
10	12.46 ± 6.02	13.64 ± 3.36	13.50 ± 2.63	17.99 ± 4.56
20	12.25 ± 3.06	13.70 ± 4.35	13.91 ± 4.09	18.81 ± 3.36
30	11.91 ± 4.12	15.36 ± 3.25	14.12 ± 6.15	17.36 ± 5.65
40	11.98 ± 3.65	15.50 ± 3.69	14.53 ± 2.51	15.57 ± 3.45
50	14.67 ± 4.12	15.22 ± 4.15	14.33 ± 4.15	14.53 ± 2.98
60	14.39 ± 5.02	14.67 ± 4.27^▲^	7.90 ± 3.39	15.09 ± 5.23^▲^
70	14.46 ± 6.35	14.39 ± 5.23	14.74 ± 3.36	15.22 ± 4.15
80	14.88 ± 5.78	15.43 ± 6.53	14.60 ± 2.25	14.95 ± 3.69
90	14.33 ± 3.26^▲^	17.85 ± 5.36^▲^	13.91 ± 2.96^▲^	15.50 ± 6.15^▲^
100	14.19 ± 6.60	18.06 ± 4.98	13.36 ± 4.51	10.87 ± 5.23
110	14.19 ± 4.97	17.69 ± 5.33	13.29 ± 5.21	18.26 ± 4.68
120	14.36 ± 1.93^▲^	17.86 ± 4.12^▲^	13.84 ± 2.36^▲^	17.99 ± 2.31^▲^
*P*	0.002			

▲*P* < 0.05 versus baseline. Baseline: the blood flow before treatment. UK: urokinase alone; US + M + UK: ultrasound, non-targeted microbubbles and urokinase; R + UK: RGDS-targeted microbubbles plus urokinase; US + R + UK: ultrasound, RGDS-targeted microbubbles and urokinase.

### Histopathological evaluation

**Figure 4:**
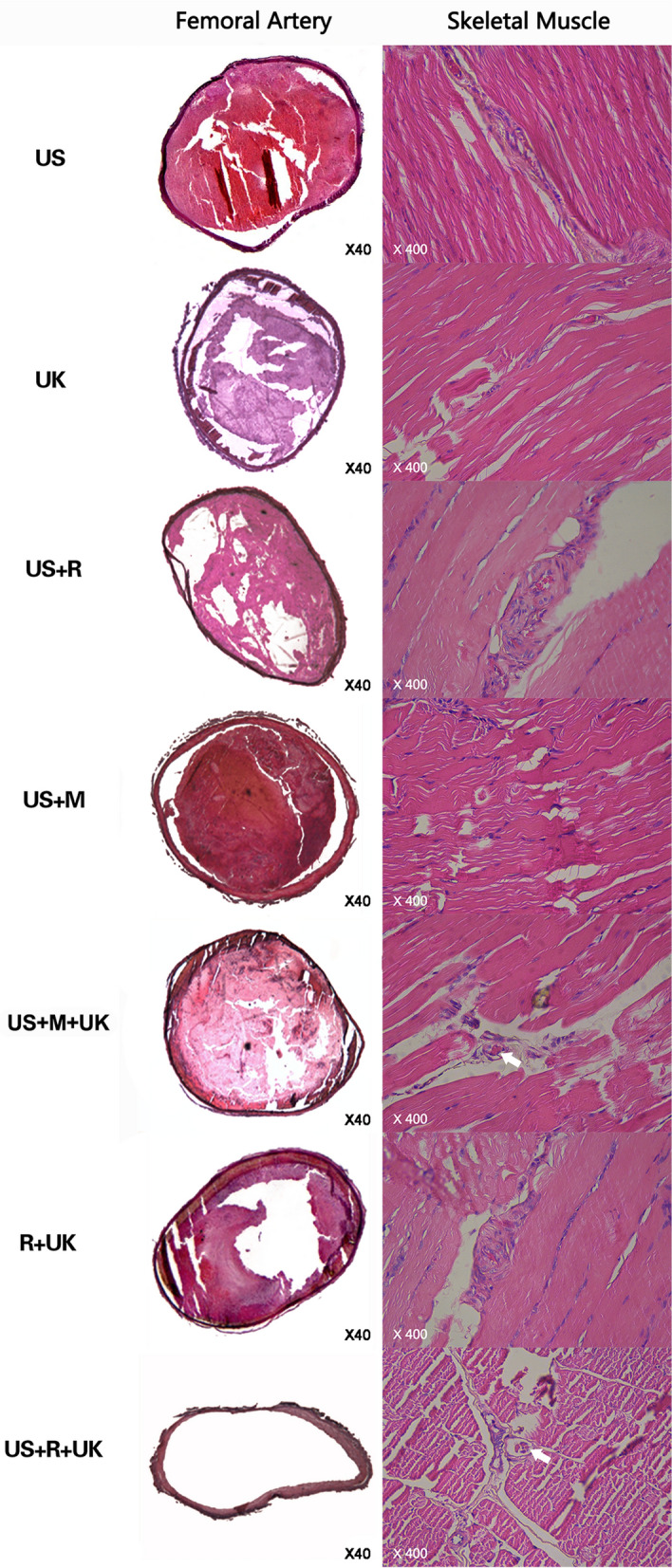
Rabbit femoral arteries stained by hematoxylin and eosin (× 40). Histological examination by H&E staining showed that the vessel was filled with thrombus in the US, UK, US + M, US + R, and US + M + UK groups, and these were not completely dissolved. Platelets appeared granular and non-dense. In the R + UK group, the thrombi were partially dissolved, with no apparent boundaries, platelets were liquefied, and massive numbers of adherent neutrophils were observed. Complete dissolution of the thrombi and recanalization of the femoral arteries were observed in the US + R + UK group. The contralateral control arteries showed no thrombi. Skeletal muscle Staining (× 400) SM: skeletal muscle. No micro thrombosis was found in skeletal muscle in the US, UK, US + M, US + R, and R + UK groups. Micro thrombosis was present in skeletal muscle microvessel in the US + R + UK and US + M + UK groups.

H&E staining showed that the vessel was filled with thrombus in the US, US + R, UK, US + M, and US + M + UK groups. Platelet beams appeared granular and non-dense, which indicated that the thrombi were partially dissolved. In the R + UK group, thrombi were partially dissolved, without apparent boundaries. Massive adherent neutrophils were observed in the R + UK group. Complete dissolution of the thrombi and recanalization of the femoral arteries was achieved in the US + R + UK group (Fig. 4).

**Figure 5:**
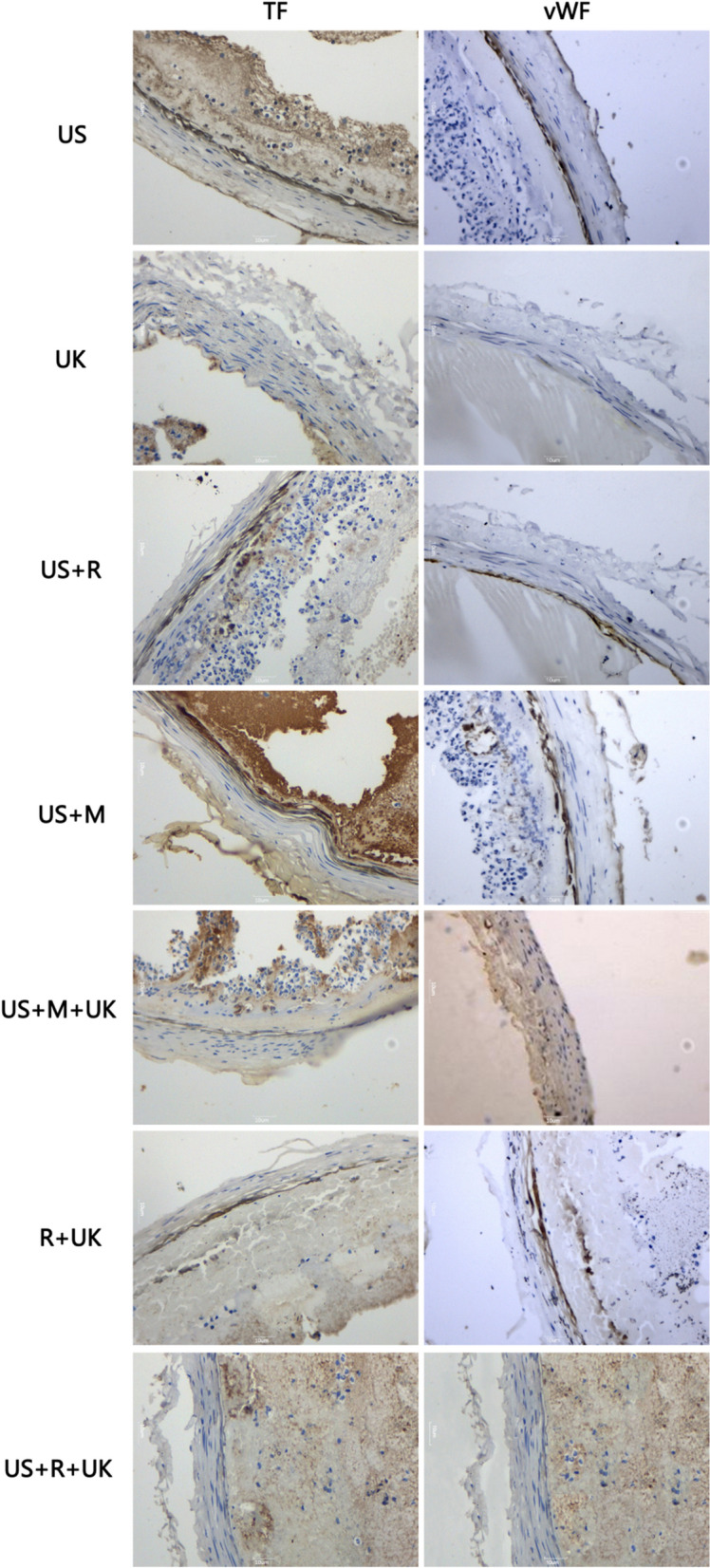
Expression of tissue factor (TF) and von Willebrand factor (vWF): US + R, UK, US + M, US groups stained positive for TF. Cytoplasm coloration, yellowish-brown granules were deposited in vascular endothelial cells. US + M + UK, R + UK, US + R + UK groups stained negative for TF. There was no deposition of yellowish-brown granules. US + R, UK, US + M, US stained positive for vWF. Cytoplasm coloration, yellowish-brown granules were deposited in vascular endothelial cells. US + M + UK, R + UK, US + R + UK groups stained negative for vWF. There was no deposition of yellowish-brown granules.

Moreover, the US, US + M, R + UK, and US + R groups stained positive for TF and vWF (Fig. 5). In contrast, the US + M + UK, UK, and US + R + UK groups were negative for TF and vWF (Fig. 5).

**Figure 6:**
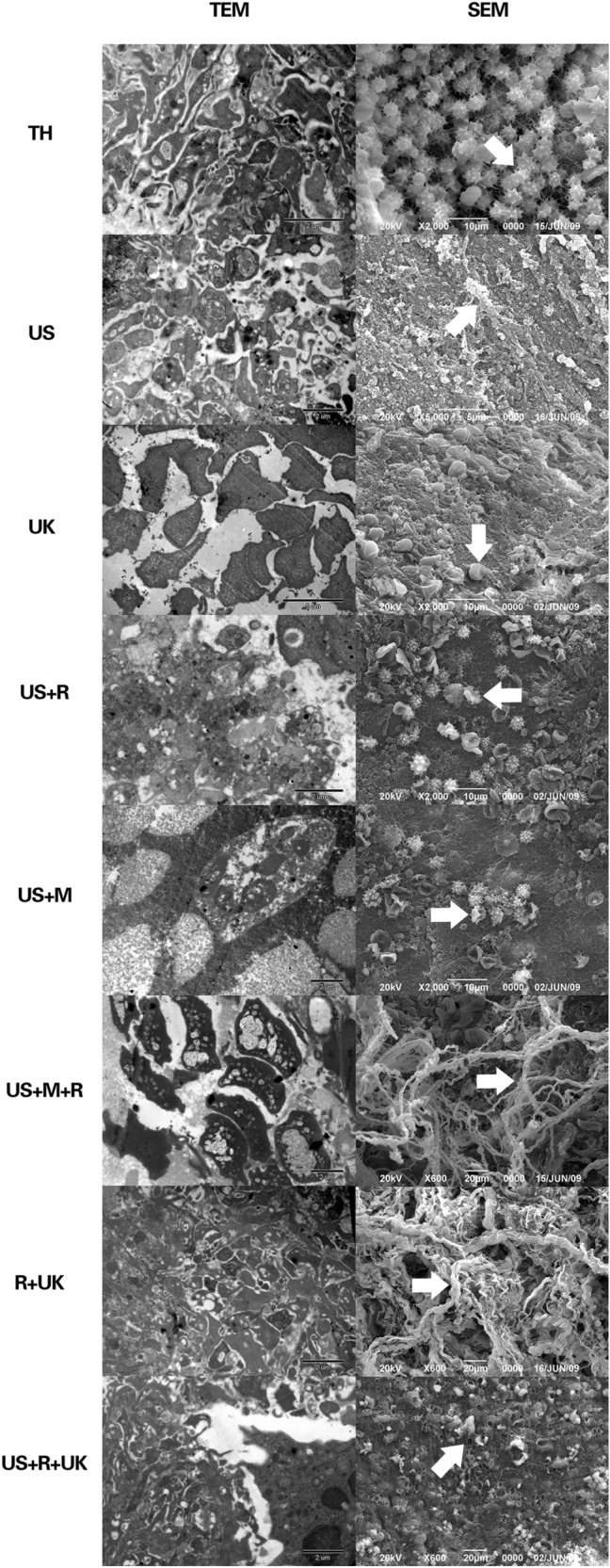
Thrombolytic effects were analyzed by SEM and TEM. In the untreated thrombus group, fibrin threads were interwoven into a net that spread over erythrocytes and platelets. The surface of the platelets had pseudopodia and showed active function. The solid white arrow indicates activated platelets. In the US group, fibrous proteins were thin, few and scattered. a few platelets were visible and regional thrombi were detached from the surface of the vessel intima. The solid white arrow indicates a fiber net fragment. In the UK group, fibrous proteins were thin. No local network was visible. Dot electron-dense patches that had been dissolved were visible, and fewer erythrocytes and platelets could be seen. The solid white arrow indicates inactivated platelets. In the US + R group, fibrous proteins were generally thin. Regional thrombi were dissolved into granules having fine sand appearance. The solid white arrow indicates inactivated platelets. In the US + M group, fibrous proteins were thin and regional thrombi were dissolved into granules having fine sand appearance. The solid white arrow indicates inactivated platelets. In the US + M + UK group, thin and loose fibrous proteins were attached to the thick and bunched collagenous fibers. The majority of electron-dense patches were ruptured and dissolved. The solid white arrow indicates a fiber net. In the R + UK group, fibrous proteins were thin, part of the structure of the fibrous protein net was not connected, and regions of fibrous proteins were broken down into particles resembling sand grains. The solid white arrow indicates a fiber net. In the US + R + UK group, most of the fibrous proteins were dissolved into particles resembling sand grains. The network structure of fibrous proteins had disintegrated. The cavities where blood cells had detached were visible in several places. The solid white arrow indicates debris.

Evaluation of the surface structure of the thrombi was analyzed by SEM. In the US, US + R, US + M, UK, US + M + UK, and R + UK groups, fibrous proteins were thin, whereas, in the US + R + UK group, most of the fibrous proteins were dissolved into particles resembling sand grains, the network structure of fibrous proteins had disintegrated, and cavities containing detached blood cells were visible in several places (Fig. 6).

**Figure 7:**
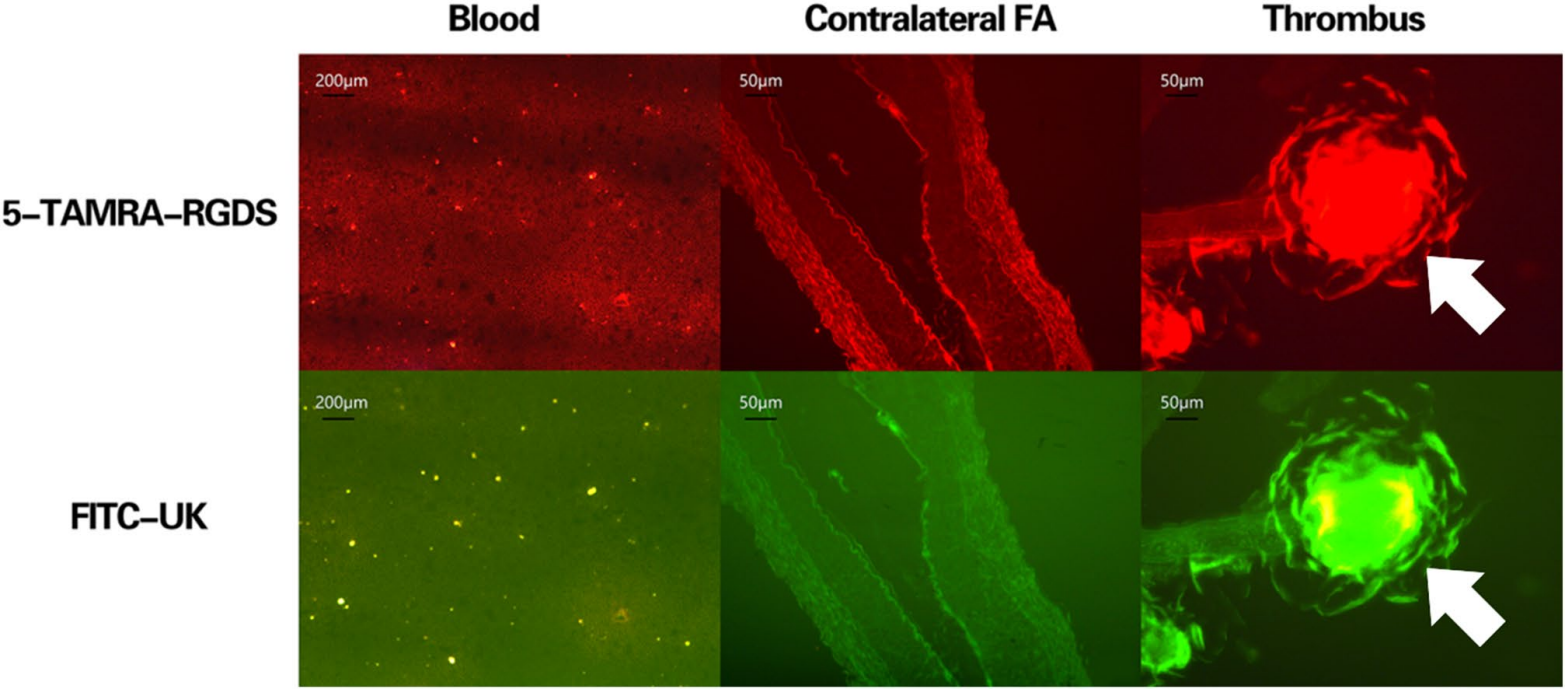
Fluorescence microscopy of microbubbles at the thrombus site, in the contralateral femoral artery, and in blood. Fluorescence microscopy showed that the TAMRA- and FITC-conjugated microbubbles accumulated (yellow clumps) at the thrombus site, with minimal signal in the contralateral femoral artery and in the blood.

SEM showed that in the US, US + M, and US + R groups, the platelets were irregular in shape and that they were scattered, with partly fragmented local aggregations. In the R + UK, US + M + R, and US + R + UK groups, most thrombi were dissolved, and plenty of degraded fragments were visible (Fig. 6).

Fluorescence microscopy showed that the TAMRA- and FITC-conjugated microbubbles accumulated at the thrombus site, with minimal signal in the contralateral femoral artery and in the blood (Fig. 7).

### Skeletal muscle staining and complications

No injuries or bleeding of soft tissues were observed in any samples. One instance of each was found in the R + UK and US + R + UK groups, and a total of two cases in the R + UK group were observed with microthrombosis in skeletal muscle (Fig. 4). There were no bleeding complications, but re-embolization of the microcirculation was observed.

## Discussion

In this study, the combination therapy in the targeted microbubble groups (R + UK and US + R + UK), enhanced thrombolysis and resulted in complete recanalization by 120 min after thrombi onset. This study suggests a potential alternative thrombolytic therapy for clinical application in the hope to reduce complications in patients.

Data from the present study revealed that US combined with RGDS targeted-microbubbles and urokinase (R + UK and US + R + UK) were more effective in achieving thrombolysis than using untargeted microbubbles. This is consistent with our previous study^33^. As shown by SEM and TEM, the treatment effect was closely related to the destruction of the thrombus structure. This was possibly caused by the increase in the local accumulation of targeted microbubbles and urokinase. As reported elsewhere, the oscillation of vapor- and/or gas-filled bubbles in a liquid under the influence of an acoustic field plays a sonothrombolysis role in driving a fibrinolytic agent into the fibrin matrix of the thrombus^34–36^. Consequently, it affects biological tissues via a bubble or cluster of bubbles that are stimulated into action by an acoustic field^37,38^.

In the present study, through continuous blood flow monitoring, we observed that targeted microbubbles caused a wave of a certain intensity and amplitude resonance (R + UK group), and the resonance was intensified by US. When microbubbles generate a large quantity of cavitational energy during collapse^39^, effective dissolution of thrombi and recanalization can be achieved. The recanalization, however, presented only after such a resonance curve appeared, indicating interactions between US and the RGDS-microbubbles during the thrombolysis process. An in vitro study from Gupta et al.^39^ reported that non-targeted bubble liposomes showed negligible enhancement of US clot disruption, which supports our findings. Data from the present study also indicated that US itself was capable to damage thrombus, primarily by distracting the fibrin matrix. This is very important for thrombolysis therapy because US, in damaging the matrix of the thrombus, can avoid the fiber proteins and platelets from re-assembling, thus, reducing the chances of re-occlusion.

Urokinase is irreplaceable in thrombolytic therapy. US induce numerous microcavities, which is specific to the microbubble-treated thrombus, and lead to thrombolysis through cavitation and resonance effect. However, when urokinase is not used, the resonance effect of US and targeted microbubbles is conditional. The number of microbubbles reached a certain threshold, in which, whether US alone or US combined with targeted microbubble (US + R) or no-targeted microbubble (US + M) failed to achieve complete recanalization. SEM and TEM results indicated that the fibrous proteins were mainly dissolved by urokinase, and the effects of urokinase were further strengthened by US or microbubble.

One issue worth to mention is that without urokinase (such as in the US + M group), the thrombus still can be dissolved and recanalization achieved (regardless of re-occlusion). This is perhaps due to that US can immediately change the fibrin structure, generating penetration of tissue plasminogen activator into the thrombus and absorption of ultrasound energy, which in turn, causes an increase in temperature, thereby accelerate the enzymatic hydrolysis of fibrin^31,40,41^.

Blood flow indicated that when urokinase concentration dropped to its lowest level, the blood flow returned to peak level, indicating an increase in the number of urokinase binding sites, which resulted from the US effect and rupture of targeted-microbubbles, thereby, enabling an accelerating thrombolysis with significant consumption of urokinase^42^. This observation is supported by an in vitro study by Francis et al.^22^, where exposure to ultrasound increased the uptake of recombinant tissue plasminogen activator (rt-PA) into blood clots and with deeper penetration. SEM and TEM results from the present study showed degraded fragments and degradation of high electron-dense particles in the US + M + UK, UK, R + UK, and US + R + UK groups. This could be due, as reported by Sutton et al., to US causing axial fluid to speed up, resulting in the phenomenon of acoustic streaming, which creates high velocity gradients at the thrombus surface. This facilitates to break up the thrombus as well as enhance the exposure of fibrin to fibrinolytic agents in a mechanical way^43^.

Another mechanism by which US + R + UK affects biological tissues is possibly through US inhibition of TF and vWF expression. TF and vWF staining were negative in the US + M + UK, and US + R + UK groups, but positive in the R + UK groups. Interestingly, in the R + UK group, even recanalization was achieved, but the fiber proteins and platelets re-assembled, resulted re-occlusion, suggesting that without US, blood clots inside the fibrous network may not be fully destroyed and, therefore, the thrombi were re-assembled. The von Willebrand factor (vWF) is highly expressed in thrombi, which are mainly composed of platelets and fibrins^44^. The glycoprotein vWF serves as an anti-hemophilic factor carrier and a platelet-vessel wall mediator at the same time in the blood coagulation system^45,46^. The activation of the extrinsic coagulation pathway triggered by exposure of tissue factor (TF) to flowing blood, is one of the key events responsible for thrombus formation after plaque rupture. In acute myocardial infarction, aggregation of TF-mediated platelet plays a key role in thrombogenesis^47,48^. The coagulation cascade activated by TF and vWF represents an important determinant of intravascular thrombus generation.

The RGDS is present in fibronectin and in other cell adhesive ECM proteins, where it mediates the binding of different integrins with different specificity^49^. Integrins are involved in some cancers, in angiogenesis, and in coagulation^49^. The RGD sequence is also a specific recognition and binding site of platelet membrane glycoprotein IIb/IIIa (GP IIb/IIIa) receptor^38^. RGD-modified liposomes are able to target and bind to the activated platelets, resulting in a high amount aggregation of platelets^39^, suggesting the feasibility of developing a drug delivery system targeting platelet and being anti-thrombogenic. Newly developed microbubbles containing bioconjugate ligands to RGD can bind to platelets specifically. Thrombolysis by the combined use of ultrasound and microbubbles involves microbubble cavitation that brings microbubbles in close proximity to the thrombus^40^. Hence, the RGDS-containing microbubbles will predominantly accumulate where integrins and GP IIb/IIIa receptors are mostly present, but it is true that they may also bind elsewhere. Nevertheless, the microbubbles will be burst only in the presence of ultrasound, which is targeted at the thrombus site, leaving the other microbubbles to disappear naturally. In addition, the urokinase will be predominantly delivered at the thrombus site, with a small amount delivered systemically, which is not worst than urokinase injection.

As far as we know, the present study is the first time to show that diagnostic US, targeted-microbubbles, and urokinase, when combined with the acoustic vibration method, exhibited a synergistic effect. Moreover, the synergism was demonstrated to achieve complete recanalization of the femoral artery in a rabbit model. Diagnostic US can destroy the fiber network structure, promote the release of targeted-microbubbles (which has the advantage to accumulate at the thrombus), and destroy and invade the thrombus through the cavitation and resonance effects. Driving the fibrinolytic agent (urokinase) into the inner fibrin matrix of the thrombus simultaneously inhibited the expression of TF and vWF, reducing the bridges among platelets. In conclusion, combined US, targeted microbubbles, and urokinase achieves a complete recanalization of the femoral artery and prohibited re-occlusion.

## Supplementary information

Supplementary file 1.

## Data Availability

The datasets used and/or analyzed during the current study are available from the corresponding author on reasonable request.
