# Author Correction: A thrombolytic therapy using diagnostic ultrasound combined with RGDS-targeted microbubbles and urokinase in a rabbit model

**DOI:** 10.1038/s41598-020-72445-1

**Published:** 2020-09-22

**Authors:** Lina Guan, Chunmei Wang, Xue Yan, Liyun Liu, Yanhong Li, Yuming Mu

**Affiliations:** Department of Echocardiography, First Affiliated Hospital of Xinjiang Medical University, Urumqi, Xinjiang People’s Republic of China

Correction to: *Scientific Reports* 10.1038/s41598-020-69202-9, published online 27 July 2020

This Article contains an error in Figure 3, where the scale of the x-axis of the panel “R + UK” was omitted. The correct Figure 3 appears below as Figure  1.

**Figure 1:**
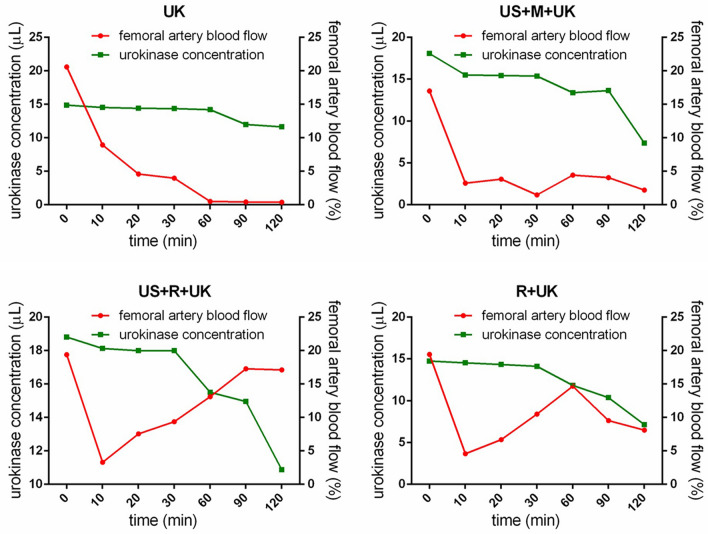
Urokinase concentration and blood flow. The urokinase concentration of the R + UK and US + R + UK group dropped to the lowest point at 60 and 100 min, respectively, while the blood flow peaked at the same time points. UK: urokinase alone; US + M + UK: ultrasound, non-targeted microbubble and urokinase; R + UK: RGDS-targeted microbubble plus urokinase; US + R + UK: ultrasound, RGDS-targeted microbubble and urokinase. Squares indicate the serum concentration of urokinase. Circles indicate the femoral artery blood flow.

