# Supplementary material for: A thrombolytic therapy using diagnostic ultrasound combined with RGDS-targeted microbubbles and urokinase in a rabbit model
Source: Sci Rep. 2020 Jul 27;10:12511. doi: 10.1038/s41598-020-69202-9 (PMC7385658; doi:10.1038/s41598-020-69202-9)
Supplement: Supplementary file 1 [file 41598_2020_69202_MOESM1_ESM.pdf]

A thrombolytic therapy using diagnostic ultrasound combined with RGDS-targeted microbubbles and urokinase in a rabbit model

Running title: A new thrombolytic therapy in a rabbit model

Lina Guan, MD, Chunmei Wang, MS, Xue Yan, MS, Liyun Liu, MD, Yanhong Li, MS, Yuming Mu, MD*

Department of Echocardiography, First Affiliated Hospital of Xinjiang Medical University, Urumqi, Xinjiang, P.R. China

***Corresponding Author:**

Yuming Mu, MD

Department of Echocardiography, First Affiliated Hospital of Xinjiang Medical University, Urumqi, Xinjiang, P.R. China

Tel: +86-13009639971

Fax: +86-21-57643271

E-mail: yumingmu@yeah.net

Supplementary Figure S1.

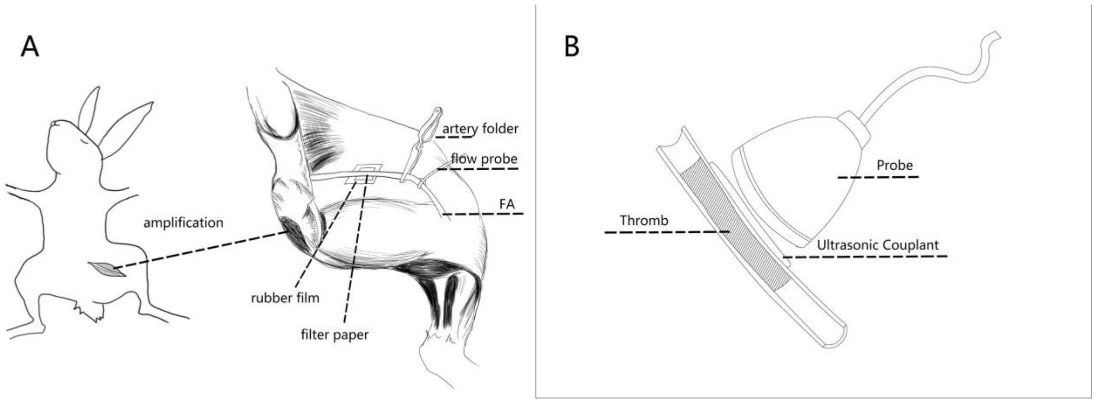

Schematic image of rat model for acute femoral artery embolism.
